# Podoplanin enhances lung cancer cell growth *in vivo* by inducing platelet aggregation

**DOI:** 10.1038/s41598-017-04324-1

**Published:** 2017-06-22

**Authors:** Kenichi Miyata, Ai Takemoto, Sakae Okumura, Makoto Nishio, Naoya Fujita

**Affiliations:** 1 Division of Experimental Chemotherapy, The Cancer Chemotherapy Center, Japanese Foundation for Cancer Research, 3-8-31, Ariake, Koto-ku, Tokyo, 135-8550 Japan; 2 Department of Computational Biology and Medical Sciences, Graduate School of Frontier Sciences, The University of Tokyo, 5-1-5, Kashiwanoha, Kashiwa-shi, Chiba, 277-8561 Japan; 3 Thoracic Oncology Center, Cancer Institute Hospital, Japanese Foundation for Cancer Research, 3-8-31, Ariake, Koto-ku, Tokyo, 1350-8550 Japan; 4 The Cancer Chemotherapy Center, Japanese Foundation for Cancer Research, 3-8-31, Ariake, Koto-ku, Tokyo, 135-8550 Japan

## Abstract

Podoplanin/Aggrus, known as a platelet aggregation-inducing factor, is frequently overexpressed in lung squamous cell carcinomas (LSCC) and glioblastomas among other tumours, and its expression has been reported to be correlated with poor prognosis. However, the contribution of podoplanin to malignant progression has been elusive. Here we demonstrate that in podoplanin-positive LSCC cells, their growth was abrogated by podoplanin knockout *in vivo* but not *in vitro*. Conversely, ectopic expression of podoplanin promoted cell growth *in vivo* and facilitated intratumoral platelet activation. Consistently, LSCC cells evoked podoplanin-mediated platelet aggregation (PMPA), and the releasates from platelets during PMPA promoted the growth of LSCC cells *in vitro*. Phospho-receptor-tyrosine-kinase array analysis revealed that epidermal growth factor receptor (EGFR) phosphorylation of LSCC cells was responsible for the growth promotion induced by platelet releasates. Treatment with an antiplatelet agent or podoplanin-neutralizing antibody depressed the growth of an LSCC tumour xenograft via suppression of EGFR phosphorylation. These results suggested that podoplanin in LSCC enhanced cell growth by inducing PMPA *in vivo* and contributed to malignant progression.

## Introduction

A platelet is anucleate blood cell derived from megakaryocytes in bone marrow and has a significant role in hemostasis, angiogenesis, vascular integrity, and wound healing^1^. The platelet contains many growth factors in its granules and releases growth factors during platelet activation^2^. Platelets extravasate into tumour microenvironments because neovasculature is leaky, and they then interact with tumour cells^3–5^. Tumour cells can evoke tumour cell-induced platelet aggregation (TCIPA), and growth factors and cytokines released from platelets contribute to tumour progression during TCIPA^6–8^. These reports suggest that the platelets in cancer facilitate tumour growth and malignant progression.

Podoplanin, also known as Aggrus or T1alpha, is a type-I transmembrane sialoglycoprotein^9, 10^ expressed in squamous cell carcinoma, glioblastoma, osteosarcoma, bladder cancer, mesothelioma and seminoma^11–15^. It has been reported that podoplanin interacts with C-type lectin-like receptor 2 (CLEC-2) in platelets and induces podoplanin-mediated platelet aggregation (PMPA). PMPA is essential for blood-lymphatic separation during development^16, 17^, and sphingosine-1-phosphate released from platelets during PMPA maintain the integrity of high endothelial venules during immune responses^18^. In contrast, podoplanin expressed in tumour cells also induces platelet aggregation (PMPA) and facilitates hematogenous dissemination^9, 19, 20^. In addition, it has been shown to be expressed in circulating tumour cells^21^, in tumour-initiating cells^22^ and on the leading edge of tumour cells^23, 24^, and its high expression correlated with poor prognosis in patients with glioblastoma and lung squamous cell carcinoma (LSCC)^25, 26^. It is also involved in tumour progression^27, 28^; however, a detailed mechanism explaining its role in tumour progression has not been elucidated.

In this study, to elucidate the mechanism underlying the role of podoplanin in tumour progression, we knocked out or ectopically expressed podoplanin in lung cancer cells. Interestingly podoplanin promoted cell growth *in vivo* but not *in vitro*. We found out that LSCC cells evoked PMPA and the releasates from platelets during PMPA involving epidermal growth factor (EGF) stimulated growth of LSCC cells via EGF Receptor (EGFR) phosphorylation *in vitro*. In addition, administration of antiplatelet agent or podoplanin-neutralizing antibody suppressed the growth of LSCC tumour xenografts by inhibiting EGFR phosphorylation *in vivo*. These results revealed for the first time a novel mechanism that podoplanin in LSCC contributed to tumour progression *in vivo*.

## Results

### Involvement of podoplanin in lung tumour growth *in vivo*

**Figure 1:**
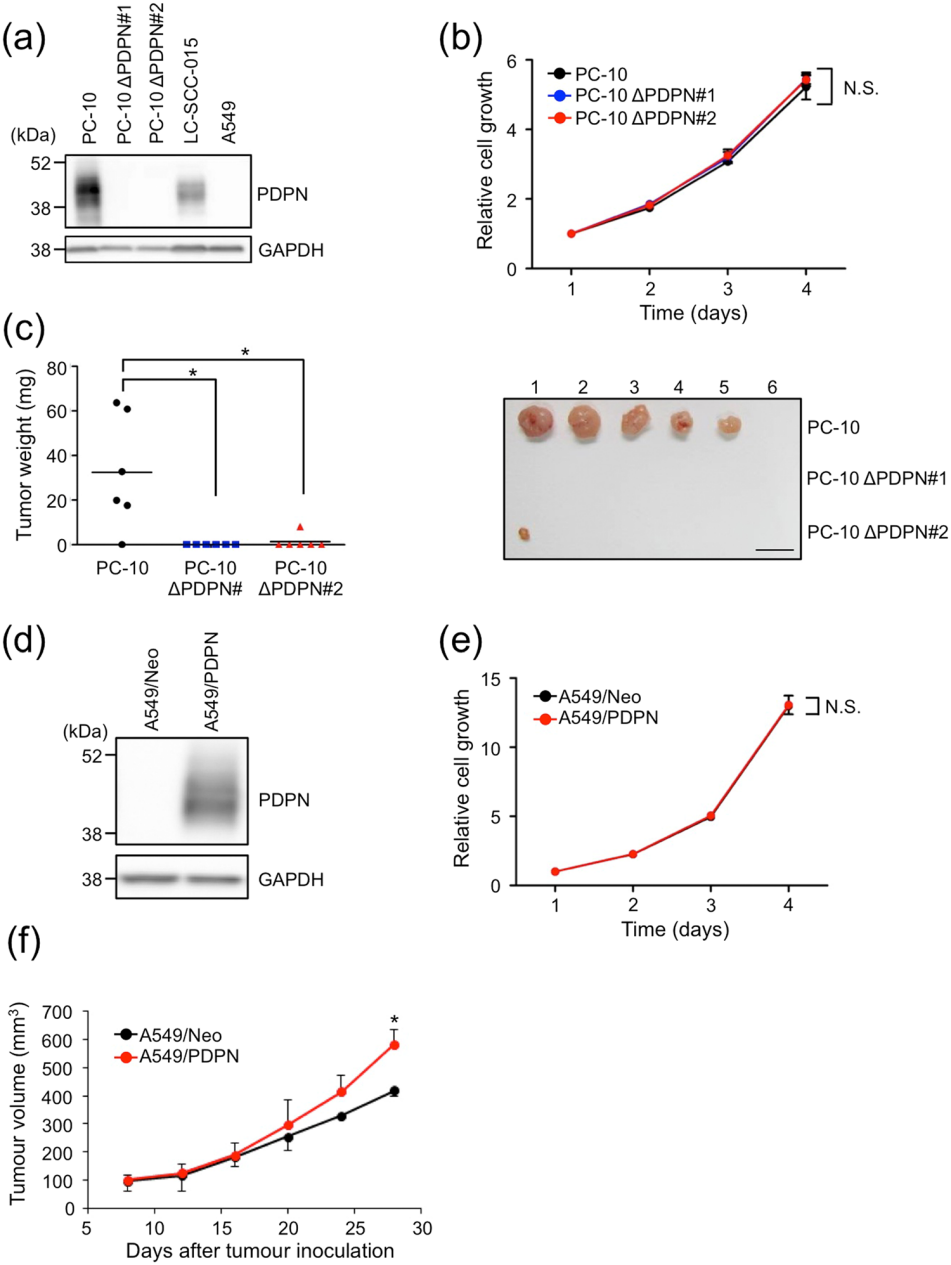
Podoplanin expression contributes to *in vivo* tumour growth but not *in vitro* cell growth. (**a**) Western blot analysis of podoplanin expression. The cell lysates of PC-10, podoplanin-knockout PC-10 (PC-10 ΔPDPN#1 and PC-10 ΔPDPN#2), SCC-015 and A549 cells were electrophoresed and immunoblotted with antibodies to podoplanin (PDPN) or GAPDH. Multiple exposure images of full-length blots were presented in Supplementary Fig. S8. (**b**) Role of podoplanin expression in cell growth in PC-10 cells *in vitro*. The *in vitro* cell growth in PC-10 and podoplanin-knockout PC-10 (PC-10 ΔPDPN#1 and PC-10 ΔPDPN#2) cells was estimated using CellTiter-Glo luminescent cell viability assay reagent. Relative cell growth was normalized to the luminescence on day 1. All data are shown as means ± SD of triplicate experiments. N.S.; Not significant by Mann–Whitney *U*-test. (**c**) PC-10 and podoplanin-knockout PC-10 (PC-10 ΔPDPN#1 and PC-10 ΔPDPN#2) cells (1 × 10^7^ cells) were subcutaneously injected into nude mice (N = 6). After 23 days, the tumours were extracted and tumour weights were measured (Left). *P < 0.05 by the Mann–Whitney *U*-test. The extracted tumours are shown (Right, scale bar: 10 mm) (**d**) Cell lysates from A549 cells that had been transfected with pcDNA3-mock (A549/Neo) or pcDNA3-podoplanin (A549/PDPN) plasmid were immunoblotted with antibodies to podoplanin (PDPN) or GAPDH. Multiple exposure images of full-length blots were presented in Supplementary Fig. S8. (**e**) *In vitro* cell growth in A549/Neo and A549/PDPN cells was estimated using CellTiter-Glo luminescent cell viability assay reagent. Relative cell growth was normalized to the luminescence on day 1. All data are shown as means ± SD of triplicate experiments. N.S.; Not significant by Student’s *t*-test. (**f**) A549/Neo and A549/PDPN cells (5 × 10^6^ cells) were subcutaneously injected into nude mice (N = 3). The tumour volume was measured every 4 days. *P < 0.05 by Mann–Whitney *U*-test.

Lung squamous cell carcinoma (LSCC) frequently overexpresses podoplanin, and its expression is correlated with poor prognosis^11, 26^. To analyze the contribution of podoplanin to tumour progression in LSCC, we knocked out podoplanin (PDPN) by using the CRISPR-Cas9 system^29^ in an LSCC cell line, PC-10, and confirmed knockout of podoplanin by Western blot and flow cytometric analysis (PC-10 ΔPDPN, Fig. 1a and Supplementary Fig. S1a). The PC-10 ΔPDPN cells did not express Cas9 protein (data not shown). Podoplanin-positive SCC-015 cells were established from LSCC patient as described in the Materials and Methods (Fig. 1a and Supplementary Fig. S1a). There was no difference in cell growth *in vitro* between PC-10 (parent) and PC-10 ΔPDPN cells (Fig. 1b). Interestingly PC-10 ΔPDPN cells could barely form tumours *in vivo* (PC-10 ΔPDPN#1; 0/6, PC-10 ΔPDPN #2; 1/6), though PC-10 (parent) cells did form tumours *in vivo* (5/6, Fig. 1c). We next overexpressed podoplanin in A549 cells in which podoplanin could not be detected endogenously (Fig. 1d and Supplementary Fig. S1b). Ectopic expression of podoplanin in A549 (A549/PDPN) cells did not affect cell growth *in vitro* (Fig. 1e). However, the tumour volume of A549/PDPN was increased *in vivo* (Fig. 1f). These results indicated that podoplanin contributed to *in vivo* tumour growth but not *in vitro* cell growth in PDPN-positive lung cancer cells.

### Elevated level of platelet aggregates in PDPN-positive tumours

**Figure 2:**
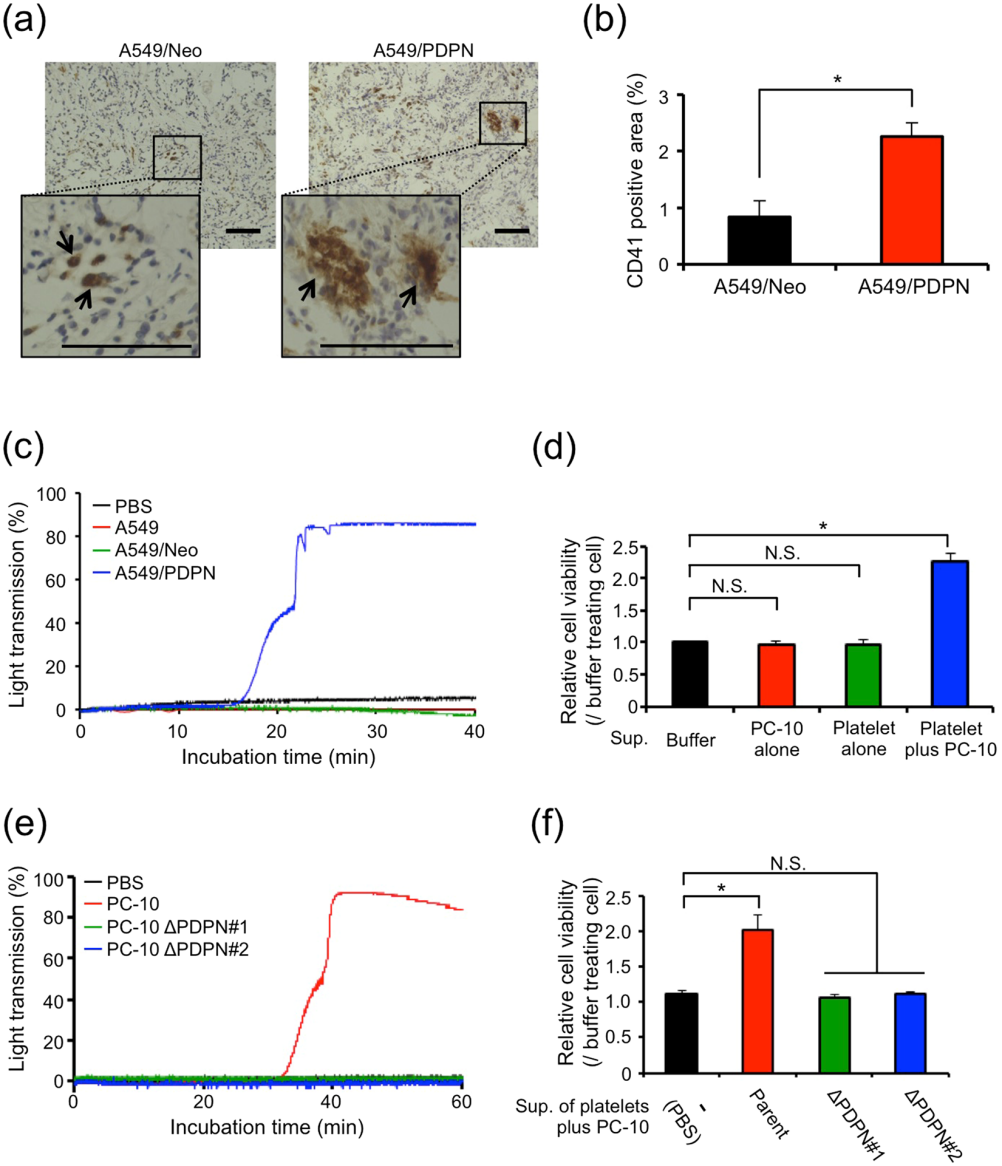
Podoplanin promotes cell growth by enhancing soluble factor secretion from platelets. (**a**,**b**) A549 cells that had been transfected with pcDNA3-mock (A549/Neo) or pcDNA3-podoplanin (A549/PDPN) plasmid (5 × 10^6^ cells) were subcutaneously injected into nude mice (N = 3). After 14 days of tumour inoculation, the tumours were extracted and stained with antibodies to CD41 (**a**). Arrows indicate platelet aggregates. Scale bar: 100 μm. The CD41-positive area was quantitated from 3 independent fields for each mouse (**b**). All data are shown as means ± SEM. (N = 3) *P < 0.05 by Mann–Whitney *U*-test. (**c**) A549/Neo or A549/PDPN cells (2 × 10^6^ cells/ml) were incubated with washed platelets (5 × 10^8^/ml) in Tyrode’s buffer containing 2% murine platelet-poor plasma and 250 μM CaCl_2_. The light transmission was measured by MCM HEMA TRACER 313 M to monitor the platelet aggregation rate. (**d**) Supernatants were collected from buffer alone, platelets alone, PC-10 alone, or platelets incubated with PC-10 cells. Then, the PC-10 cells that had been transfected with *ZsGreen* gene (PC-10/ZsG) were cultured for 72 hours in each supernatant under 0.5% FBS condition. The cell viability of the PC-10/ZsG was calculated from ZsGreen fluorescence. All data are shown as means ± SD of triplicate experiments. *P < 0.05 by Mann–Whitney *U*-test. N.S.: Not significant. (**e**) PC-10 and podoplanin-knockout PC-10 (PC-10 ΔPDPN#1 and PC-10 ΔPDPN#2) cells (1 × 10^6^ cells/ml) were incubated with washed platelets (5 × 10^8^/ml) in Tyrode’s buffer containing 2% platelet-poor plasma and 250 μM CaCl_2_. The light transmission was measured by MCM HEMA TRACER 313 M to monitor platelet aggregation rate. (**f**) Supernatants were collected from platelets incubated with PC-10 (parent), PC-10 ΔPDPN#1 or PC-10 ΔPDPN#2 cells. Then, PC-10/ZsG cells were cultured with the collected supernatants under 0.5% FBS condition. After 72 hours, the cell viability of the PC-10/ZsG was calculated from ZsGreen fluorescence. All data are shown as means ± SD of triplicate experiments. *P < 0.05 by Mann–Whitney *U*-test. N.S.: Not significant.

Because podoplanin is a platelet aggregation-inducing factor^9, 10, 19^, we focused on the activation of intratumoral platelets. Platelet aggregates were more detected in A549/PDPN tumours rather than in A549/Neo tumours (Fig. 2a,b and Supplementary Fig. S2a,b). Moreover, we also found the colocalization of platelet aggregates, detected by anti-fibrin/fibrinogen antibody, with strong PDPN staining in PC-10 tumour (Supplementary Fig. S2c). Consistently, A549/PDPN cells evoked platelet aggregation *in vitro*, but A549/Neo cells did not (Fig. 2c). Similarly PC-10 and SCC-015 cells evoked platelet aggregation, and the platelet aggregation was suppressed by anti-human podoplanin antibody, MS-1 that had neutralized the interaction of podoplanin with CLEC-2 on platelets^5^ (Supplementary Fig. S3a,c). These results indicated that podoplanin-positive LSCC cells induced podoplanin-mediated platelet aggregation (PMPA). We next examined the effect of the releasates from activated platelets during PMPA on cell growth because platelets have been shown to contain many growth factors in their granules and release growth factors during activation^8^. The releasates from platelets during PMPA were collected from the supernatant of a LSCC-platelet reactant, as described in the Materials and Methods. Interestingly, the supernatant promoted the growth of PC-10 or SCC-015 cells proportional to the supernatant concentration (Fig. 2d and Supplementary Fig. S3b,d). In support of these data, neither PC-10 ΔPDPN cells induced platelet aggregation (Fig. 2e) nor the supernatant of a PC-10 ΔPDPN-platelet reactant promoted cell growth (Fig. 2f). These results suggested that platelet aggregation induced by PDPN-positive LSCC cells was dependent on podoplanin, and the releasates from platelets during PMPA stimulated LSCC cell growth.

### Platelet-derived epidermal growth factor (EGF) partially contributed to cell growth promoted by releasates from platelets during PMPA

**Figure 3:**
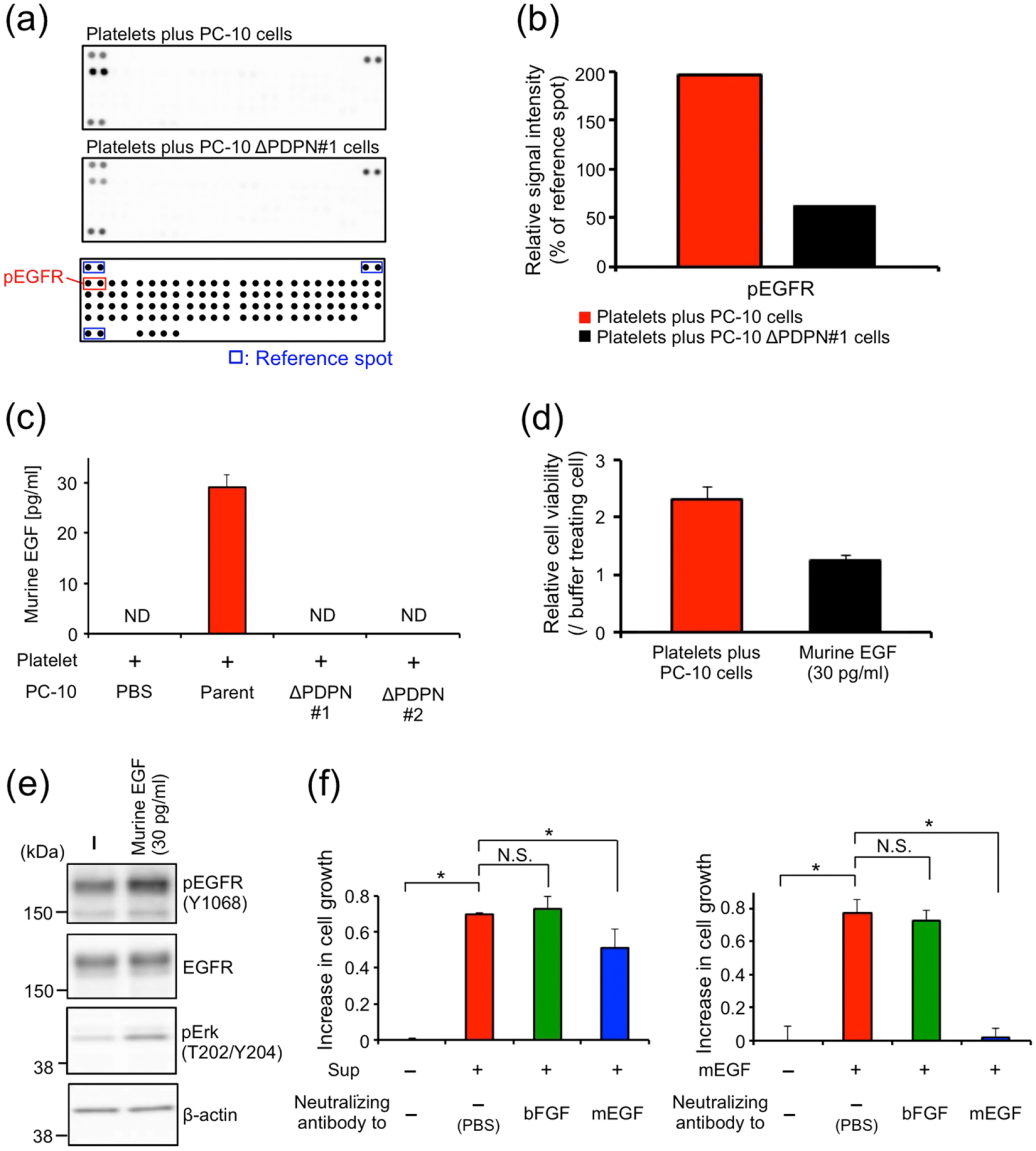
Platelet-derived murine epidermal growth factor (EGF) partially contributed to cell growth promoted by platelet releasates during PMPA. (**a**,**b**) PC-10 (parent) cells starved for 24 hours were treated with supernatants collected from platelets incubated with PC-10 (parent) or PC-10 ΔPDPN#1 cells. The treated cell lysates were used in a phospho-RTK array according to the manufacturer’s protocols. The probed membranes are shown in (**a**). The signal intensity of epidermal growth factor receptor (EGFR) phosphorylation (within red squares) was quantified by Image J software (NIH). The signal intensities of six reference spots (within blue squares) in each membrane were measured and defined as 100%. The relative intensities of duplicate spots are shown in (**b**). (**c**) Murine EGF concentration in the supernatants collected from platelets incubated with PBS, PC-10 (parent), PC-10 ΔPDPN#1 and PC-10 ΔPDPN#2 cells was measured by ELISA. ND; Not detected. All data are shown as means ± SD of triplicate experiments. (**d**) PC-10/ZsG cells were incubated with supernatant of a PC-10 (parent)-platelet reactant or 30 pg/ml murine EGF under 0.5% FBS condition. After 72 hours incubation, the cell viability of the PC-10/ZsG was calculated from ZsGreen fluorescence. All data are shown as means ± SD of triplicate experiments. (**e**) PC-10 (parent) cells starved for 24 hours were treated with 30 pg/ml murine EGF for 2.5 minutes. After ice-cold PBS wash, the cells were lysed with SDS lysis buffer, and Western blot analysis was performed. (**f**) The supernatant of a PC-10 (parent)-platelet reactant (left) or 5 ng/ml murine EGF (right) was incubated with 28.6 μg/ml anti-human bFGF or anti-mouse EGF neutralizing antibody (R&D Systems) for 1 hour in a 37 °C water bath. Then, the mixture was added to PC-10/ZsG cells. After 72 hours of cultivation under 0.5% FBS condition, the cell viability of the PC-10/ZsG was calculated from the ZsGreen fluorescence. All data are shown as means ± SD of triplicate experiments. *P < 0.05 by Mann–Whitney *U*-test. N.S.: Not significant.

To clarify how the releasates from platelets during PMPA stimulated cell growth, we analysed the receptor activation of the cells by using a phospho-receptor–tyrosine–kinase (RTK) array. The results showed that phosphorylation of epidermal growth factor (EGF) receptor (EGFR) was strongly increased by cultivation with the supernatant of a PC-10-platelet reactant relative to that of a PC-10 ΔPDPN#1-platelet reactant (Fig. 3a and b). Therefore, we performed an enzyme-linked immunosorbent assay (ELISA) to identify platelet-derived growth factors that activated EGFR. Because EGF is the only growth factor reported to be an EGFR ligand in platelets^1, 2^ as far as we know, we focused on EGF as a promoter of cell growth. Approximately 30 pg/ml of murine EGF was detected in the supernatant of a PC-10-platelet reactant (5 × 10^8^/ml), but not detected in the supernatant of a PC-10ΔPDPN-platelet reactant (Fig. 3c). This concentration of murine EGF promoted growth of PC-10 cells (approximately 1.26 times, Fig. 3d), and stimulated EGFR phosphorylation in PC-10 cells (Fig. 3e). Because the supernatant of a PC-10-platelet reactant promoted growth of PC-10 cells, approximately 2.32 times, the growth promotion by the 30 pg/ml of murine EGF was estimated at 19.7%. In agreement with these findings, murine EGF-neutralizing antibody, which neutralized increased cell growth by treatment with 1 ng/ml murine EGF (Fig. 3f, right), partially inhibited growth by the addition of the supernatant of a PC-10-platelet reactant (approximately 20%) (Fig. 3f, left). These results indicated that platelet-derived murine EGF released during PMPA stimulated growth of LSCC cells.

### EGFR-TKI treatment abrogated growth promotion by the releasates from platelets during PMPA

**Figure 4:**
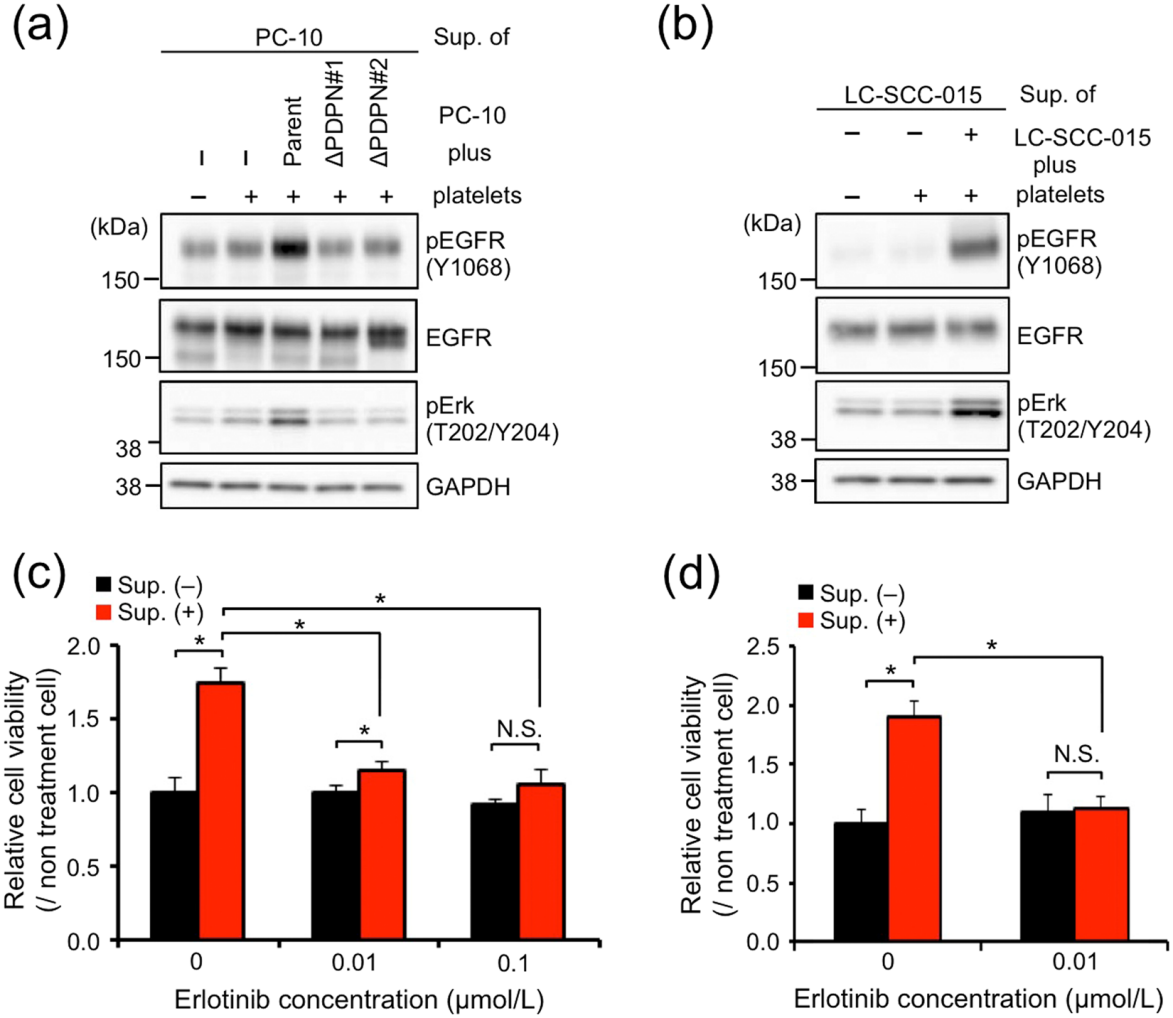
Supernatant of a PC-10-platelet reactant activated EGFR signal. (**a**) PC-10 (parent) cells starved for 24 hours were treated for 2.5 minutes at 37 °C with supernatant collected from platelets alone or platelets plus PC-10, PC-10 ΔPDPN#1, or PC-10 ΔPDPN#2 cells. After ice-cold PBS wash, the cells were lysed with SDS lysis buffer, and Western blot analysis was performed. (**b**) SCC-015 cells starved for 24 hours were treated for 2.5 minutes in 37 °C with supernatant collected from platelets alone or platelets plus SCC-015 cells. After ice-cold PBS wash, the cells were lysed with SDS lysis buffer, and Western blot analysis was performed. Multiple exposure images of full-length blots were presented in Supplementary Fig. S8. (**c**) PC-10/ZsG cells were incubated with erlotinib at the indicated concentration. After incubation for 30 minutes, PC-10/ZsG cells were treated with the supernatant of a PC-10 (parent)-platelet reactant and incubated for 72 hours under 0.5% FBS condition, followed by measuring ZsGreen fluorescence as cell viability. All data are shown as means ± SD of triplicate experiments. *P < 0.05 by Mann–Whitney *U*-test. N.S., Not significant. (**d**) SCC-015 cells were incubated with erlotinib at the indicated concentration. After incubation for 30 minutes, SCC-015 cells were treated with the supernatant of a SCC-015-platelet reactant and incubated for 72 hours under 0.5% FBS condition. Cell viability was detected by Cell Proliferation ELISA, BrdU. All data are shown as means ± SD of triplicate experiments. *P < 0.05 by Mann–Whitney *U*-test. N.S.: Not significant.

Because EGF-involved releasates from platelets during PMPA stimulated phosphorylation of EGFR (Fig. 3a and b), we next examined activation of EGFR signal. The supernatant of a PC-10 (parent)-platelet reactant activated EGFR signal, but the intensity of the EGFR signal when treated with the supernatant of a PC-10 ΔPDPN-platelet reactant was similar in extent to that of no treatment (Fig. 4a). Consistently, the supernatant of a SCC-015-platelet reactant also promoted EGFR signal (Fig. 4b). It has been proven that podoplanin itself does not contribute to the sensitivity of cells to EGF and platelet releasates (Supplementary Fig. S4). From these results, we next examined whether an EGFR-tyrosine kinase inhibitor (EGFR-TKI), erlotinib, could inhibit cell growth facilitated by the releasates from platelets during PMPA. As expected, erlotinib treatment strongly abolished growth promotion, but it did not affect basal growth of LSCC cells (Fig. 4c and d). These results indicated that the releasates from platelets during PMPA stimulated growth of LSCC cells via EGFR signal and EGFR-TKI treatment abrogated the growth promotion induced by platelet releasates.

### Podoplanin activated EGFR signal by inducing PMPA *in vivo*

**Figure 5:**
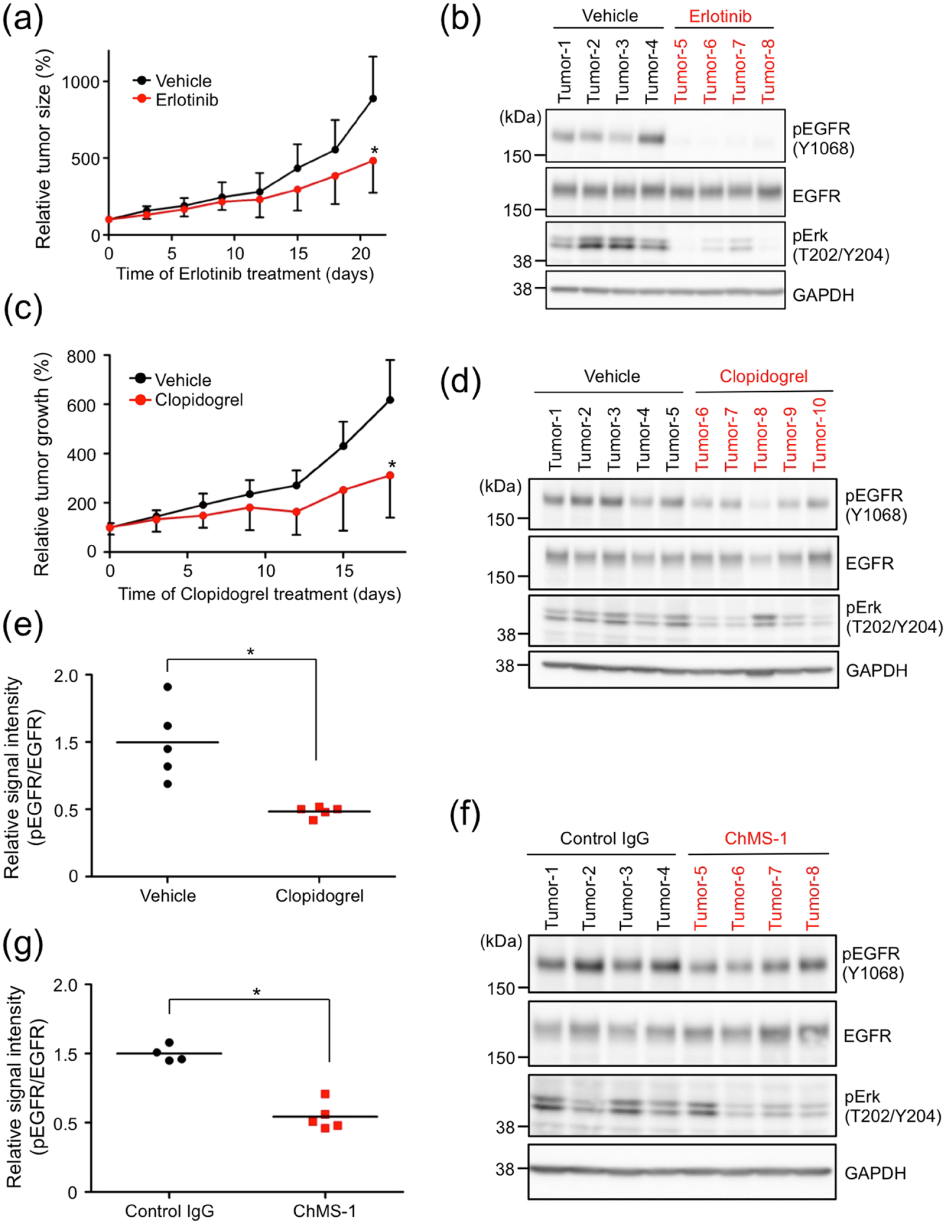
Activation of EGFR signalling induced by PMPA was suppressed by treatment of antiplatelet agent or podoplanin-neutralizing antibody *in vivo*. (**a**,**b**) PC-10 cells (5 × 10^6^ cells) were subcutaneously injected in BALB/c-nu/nu mice. After 15 days, BALB/c-nu/nu mice bearing PC-10 tumour were dosed orally with 50 mg/kg erlotinib daily. Tumour volume was measured every 3 days (**a**). All data are shown as means ± SD. (N = 4) *P < 0.05 by Mann–Whitney *U*-test. After 3 weeks, the tumours were extracted and homogenized in SDS lysis buffer. The phosphorylated-EGFR signal in the tumours was analysed by Western blot (**b**). Multiple exposure images of full-length blots were presented in Supplementary Fig. S8. (**c**–**e**) PC-10 cells (5 × 10^6^ cells) were subcutaneously injected in BALB/c-nu/nu mice. After 15 days, clopidogrel dissolved in drinking water with 0.003% HCl (equal to orally dosed 25 mg/kg/daily) was given to BALB/c-nu/nu mice bearing PC-10 tumours. Tumour volume was measured every 3 days (**c**). All data are shown as means ± SD. (N = 5) *P < 0.05 by Mann–Whitney *U*-test. After 18 days, the tumours were extracted and homogenized in SDS lysis buffer. The phosphorylation of EGFR signal in the tumours was analysed by Western blot (**d**) and the signal intensity of EGFR phosphorylation was quantified by Image J software (NIH) (**e**). *P < 0.05 by Mann–Whitney *U*-test (**f**,**g**). PC-10 cells (5 × 10^6^ cells) were subcutaneously injected into NOD/SCID mice. After 18 days, these mice were treated with 100 μg/mouse control human IgG or chimeric MS-1 (ChMS-1) antibody every week by intravenous administration as previously described^5^. After 15 days, the tumours were extracted and homogenized in SDS lysis buffer. The phosphorylated-EGFR signal in the tumours was analysed by Western blot (**f**), and the signal intensity of EGFR phosphorylation was quantified by Image J software (NIH) (**g**). (N = 4) *P < 0.05 by Mann–Whitney *U*-test.

Because EGFR-TKI treatment abrogated the growth promotion induced by PMPA *in vitro* (Fig. 4c and d), we next treated PC-10 tumour xenografts with erlotinib *in vivo*. Although erlotinib treatment did not inhibit basal growth of PC-10 cells *in vitro* (Fig. 4c), it did suppress the growth of PC-10 tumour xenografts *in vivo* and EGFR phosphorylation in the tumour (Fig. 5a and b). From these data, we speculated that PC-10 cells needed to activate platelets for outgrowth *in vivo*. To prove this hypothesis, we focused on an antiplatelet drug, clopidogrel, which could perfectly inhibit platelet aggregation induced by adenosine phosphate and could slightly inhibit it when induced by collagen (Supplementary Fig. S5a,b). These results are consistent with those of a previous report^30^. Clopidogrel treatment suppressed PMPA *in vitro* (Supplementary Fig. S5c), suppressed growth of PC-10 tumour xenografts *in vivo*, and moderately abrogated EGFR phosphorylation in the tumour xenograft (Fig. 5c–e). We previously reported that humanized chimeric MS-1 (ChMS-1) antibody suppressed PC-10 tumour growth by inhibiting PMPA induced by the interaction of podoplanin with CLEC-2 on platelets^5^. Therefore, we investigated the antitumor effects due to suppression of EGFR phosphorylation. Treatment with ChMS-1 antibody moderately inhibited EGFR phosphorylation in the tumour xenograft (Fig. 5f and g). These results suggested that podoplanin activated platelet aggregation via interaction with CLEC-2 on platelets, and the EGF-involved releasates from platelets during PMPA stimulated growth of LSCC via EGFR signalling *in vivo*.

## Discussion

LSCC is known as a high-motility cancer, and has a mortality of >400,000 people each year^31^. To develop better therapeutic strategies, clarification of the malignant mechanism in LSCC has been needed. In lung adenocarcinoma patients who have activating mutations in the EGFR, it has been reported that EGFR-TKI is an effective therapy^32^. Although activating mutations in EGFR are almost not detected in LSCC patients, it has been reported that EGFR-TKI therapy was effective for some LSCC patients; EGFR-TKI, erlotinib, and gefitinib have been approved as therapeutic agents for LSCC patients^33, 34^. The underlying mechanism, however, has not been elucidated. Our data showed that erlotinib treatment suppressed growth promotion of a supernatant of a LSCC cell-platelet reactant, but it did not inhibit basal cell growth of LSCC cells *in vitro* (Fig. 4c and d). These data suggested that podoplanin-positive LSCC activated platelets by interacting with CLEC-2 on platelets and received EGFR ligands involving EGF released from activated platelets for tumour growth *in vivo*. To examine the possibility that the growth promotive effects of a supernatant of a PC-10-platelet reactant was due to the EGFR overexpression, we analyzed the expression level of EGFR in PC-10 and SCC-015 cells, both were stimulated to grow in response to the supernatant of PDPN-mediated platelet aggregation (PMPA) (Supplementary Fig. S3b,d). We firstly compared the EGFR expression level in PC-10 and SCC-015 with that in an EGFR-overexpressing lung cancer A431 cell line and in a normal lung-derived TIG-3 cell line. As shown in new Supplementary Fig. S6a,b, EGFR expression level in PC-10 cells was close to that in EGFR-overexpressing A431 cells. EGFR expression level in SCC-015 cells was approximately equal to that in normal lung-derived TIG-3 cells. Nevertheless, SCC-015 cell growth was promoted by murine EGF (Supplementary Fig. S6c). From these results, we concluded that EGFR overexpression was not the only reason for PMPA-induced EGFR activation and tumour growth in LSCC. In this study, we focused on LSCC. It has been reported that patients with bladder carcinoma expressed EGFR and high expression correlated with poor prognosis^35^. Bladder carcinoma patients have also been shown to express podoplanin^14^. In this type of carcinoma, there is also a high possibility of the same malignant progression initiated by podoplanin. We hope to emphasize the possibility that EGFR-TKIs and/or suppressing PMPA could be effective for the PDPN-positive lung cancers and bladder carcinomas.

Erlotinib partially inhibited growth of PC-10 tumour xenografts (Fig. 5a). From this result, we considered the contribution of pathways other than EGFR signalling. Platelets contain basic fibroblast growth factor (bFGF) and vascular endothelial growth factor in their granules, and these growth factors contribute to neovascularization^2^. Tumours require neovascularization for their outgrowth, but erlotinib treatment cannot inhibit neovascularization. We think these mechanisms explain why PC-10 tumours are not perfectly suppressed by erlotinib treatment *in vivo*.

Although EGF has been shown to be the only EGFR ligand in platelets^2, 36^, the function of EGF in platelets has remained elusive. This study is the first to quantify EGF released by PMPA (Fig. 3c) and to confirm the contribution of platelet-derived EGF to cell growth (Fig. 3f). The contribution of platelet-derived EGF on cell growth was unfortunately approximately 20% (Fig. 3f). We thought that other EGFR ligands also contributed to LSCC cell growth. It has been reported that platelet contained many kinds of factors such as PDGF, bFGF, VEGF and thrombin, and released them during activation. We therefore evaluated the effect of the factors on PC-10 cell growth, but these factors could not induce the growth, though these could promote the growth of such factor-responsive cells (Supplementary Fig. S7). As other pathway, Liska and colleague have reported that hepatocyte growth factor (HGF)-activating c-MET rescued EGFR inhibition in colorectal cancer cells^37^. Since platelets contain HGF in their granules^38^, platelet-derived HGF, therefore, also may partially contribute to LSCC cell growth promoted by releasates from platelets during PMPA^2^. EGF, in addition to bFGF and transforming growth factor-β, also induces expression of podoplanin in breast cancer and oral squamous cells^23, 39^. Because these growth factors are contained in granules of platelets, they are released during PMPA, may promote tumour growth, and induce expression of podoplanin, which could activate platelets; therefore, intratumoral PMPA can initiate tumour malignant progression cycle.

Since a supernatant of LSCC-platelet reactant assists the growth of LSCC cells (Fig. 2d), we used an antiplatelet agent, clopidogrel, to investigate the effect of platelets on the growth of PC-10 tumour xenografts. Clopidogrel, which is an inhibitor targeting P2Y12, failed to perfectly inhibit platelet aggregation induced by PC-10 cells (Supplementary Fig. S5c). PMPA may need dimerization of CLEC-2 on platelets^40, 41^, followed by phosphorylation of Src and Syk kinase family members needed for activation^42^. In recent years, Navarro-Núñez *et al*. demonstrated that dasatinib, an Src/Abl inhibitor, suppressed PMPA through inhibiting the CLEC-2 signal pathway^43^. Furthermore, Chang *et al*. have shown that 2CP bound CLEC-2 and inhibited PMPA^44^. As releasates from platelets during PMPA promoted cell growth (Fig. 2f), these drugs can also suppress growth of LSCC cells *in vivo*. There is evidence that a P2Y12 inhibitor, MRS 2395, also suppressed TCIPA^45^, and clopidogrel suppressed tumour growth by inhibiting platelet aggregation^46^. Therefore, we used clopidogrel as an antiplatelet agent. Indeed, we were able to suppress growth of PC-10 tumour xenografts by clopidogrel treatment (Fig. 5c).

In general, it is known that platelets are upregulated in patients with lung cancer and that a high platelet count is correlated with poor prognosis^47, 48^. Nevertheless, no significant correlation has been observed between high platelet counts and increased risk in patients with LSCC^48^. From these reports, we hypothesized that high expression of podoplanin is important in tumour growth along with activation of platelets rather than the number of platelets. Indeed, although A549/PDPN tumours promoted tumour growth and activation of platelets in contrast to A549/Neo tumours *in vivo*, there was no difference in the number of platelets in circulation between A549/Neo-bearing mice and A549/PDPN-bearing mice (9.20 ± 0.68 × 10^9^/μl vs. 9.09 ± 0.49 × 10^9^/μl, *P* = 0.86 by Student’s *t*-test, N = 3).

Previously, we reported that a humanized chimeric anti-podoplanin antibody, ChMS-1 suppressed PC-10 tumour growth by independent antibody effector activity^5^. In this report, a PC-10 tumour treated with ChMS-1 antibody showed a decrease in EGFR signal *in vivo* (Fig. 5f and g). This finding suggested that ChMS-1 antibody suppressed EGFR signal by inhibiting PMPA. It has been shown that PMPA is important to maintaining the integrity of high-endothelial venules when lymphocytes are extravasated^18^ and to formation of lymphatic vessels during development^49^. These findings indicated that LSCC cells hijacked PMPA, which is essential in the process of homeostasis during malignant progression^5^.

In this study, we showed that podoplanin in LSCC induced platelet aggregation via interaction with CLEC-2 on platelets, and platelet releasates involving EGF promoted growth of LSCC cells by activating EGFR signalling. These findings, therefore, suggested that the interaction of podoplanin with CLEC-2 in platelets was a trigger for LSCC progression. When considering therapeutic strategies for LSCC, it is important that suppression of PMPA be achieved.

## Methods

### Cell lines

PC-10 (Immuno-Biological Laboratories, Gunma, Japan) and A549 (American Type Culture Collection) were cultured in Dulbecco’s Modified Eagle Medium (DMEM) (Sigma-Aldrich, St. Louis, MO) containing 10% foetal bovine serum (FBS) and 100 μg/ml kanamycin. A patient-derived SCC-015 cell line was established in our laboratory. A clinical lung squamous cell carcinoma specimen was obtained from a patient after providing informed consent at the Cancer Institute Hospital in the Japanese Foundation for Cancer Research (JFCR). The established patient-derived lung squamous cell carcinoma line SCC-015 cells were cultured in RPMI/F-12 medium supplemented with 15% FBS, 20 mM HEPES buffer (pH 7.5) and antibiotic–antimycotic (Nacalai Tesque, Inc., Kyoto, Japan). All the methods were carried out in accordance with relevant guidelines and regulations, and we performed all analyses following the experimental protocols approved by the Institutional Review Board (IRB) of JFCR. PC-10 cells that had stably been transfected with the *ZsGreen* gene (PC-10/ZsG) were cultured in DMEM growth medium containing 500 μg/ml of G418 (Life Technologies, Carlsbad, CA, USA), as previously described^8^. A549 was transfected with pcDNA3 plasmid (A549/Neo) or pcDNA3-PDPN plasmid^10^ (A549/PDPN), and cells that stably expressed vector were selected in 500 μg/ml G418-containing medium. A431 and HUVEC-SV40 cells were cultured in DMEM (Sigma-Aldrich) containing 10% FBS and 100 μg/ml kanamycin. BALB/3T3 were cultured in RPMI-1640 (Wako, Osaka, Japan) containing 10% FBS and 100 μg/ml kanamycin. T98G (RIKEN BRC Cell Bank, Ibaraki, Japan) cells were cultured in Eagle’s Minimum Essential Medium (Sigma-Aldrich) containing 10% FBS and 100 μg/ml kanamycin.

### CRISPR-Cas9–mediated establishment of podoplanin knockout PC-10 cells

Guide DNAs (5′-CACCGGTAGTCTCAGTGTCATCTTC-3′ and 5′-AAACGAAGATGACACTGAGACTACC-3′) and (5′-CACCGGAAGGCGGCGTTGCCATGCC-3′ and 5′-AAACGAAGATGACACTGAGACTACC-3′) were annealed and formed double-strand DNA, respectively. The double-strand guide DNAs were cloned into pSpCas9n(BB)-2A-Puro (PX462) as previously described^29^. Both plasmids were co-transfected into PC-10 cells by using Lipofectamine 2000 reagent (Life Technologies) according to the manufacturer’s protocols. Transfected cells were selected by treatment with 1 μg/ml puromycin and podoplanin knockout clones were selected by Western blot and flow cytometric analysis.

### Western blot analysis

Cell pellet and tumour fragments were lysed in lysis buffer (0.1 M Tris–HCl pH 7.5, 10% glycerol and 1% sodium dodecyl sulfate), boiled for 5 minutes and centrifuged for 10 minutes (15,000 rpm). All protein concentrations were determined by BCA Protein Assay Reagent (Pierce, Rockford, IL, USA). Each cell lysate (10 μg) was electrophoresed on Extra PAGE One Precast Gel 5–20% (Nacalai Tesque) and transferred onto PVDF membrane (Millipore Corp., Billerica, MA). After blocking with 5% skim milk (Megumilk, Tokyo, Japan) or 5% bovine serum albumin (Nacalai Tesque), the membrane was treated with primary antibodies to podoplanin (FL-162; Santa Cruz Biotechnology, CA, USA), EGFR (phospho Y1068) (Abcam, Cambridge, MA, USA), GAPDH (Millipore), β-actin (clone, AC-15, Sigma-Aldrich), EGFR and pErk (Cell Signaling Technology, Boston, MA, USA). After treating with an ECL Prime Western Blotting Detection reagent (GE Healthcare), we used LAS-3000 mini (Fujifilm, Tokyo, Japan) to detect chemiluminescence signals.

### Flow cytometric analysis

Harvested cells were incubated with 1 μg/ml mouse anti-human podoplanin antibody MS-1^5^ or control mouse IgG2a antibody (Sigma-Aldrich), followed by incubation with 4 μg/ml of Alexa Fluor 488-conjugated anti-mouse IgG (H + L) (Thermo Fisher Scientific, Waltham, MA, USA). Flow cytometric analysis was performed on a Cytomics FC500 Flow Cytometry system (Beckman Coulter, CA, USA). Date analysis was performed by using FlowJo software (Tree Star Inc.).

### Platelet aggregation assay

Murine washed platelets were collected from Jcl:ICR mice, as previously described^5, 50^. Before the experiments, 5 × 10^8^/ml platelets were mixed with 250 µM CaCl_2_ containing 2% murine platelet-poor plasma in modified Tyrode’s buffer (137 mM NaCl, 11.9 mM NaHCO_3_, 0.4 mM Na_2_HPO_4_, 2.7 mM KCl, 1.1 mM MgCl_2_ and 5.6 mM glucose, pH 7.9). After adding PC-10 (1 × 10^6^ cells/ml), SCC-015 (2.5 × 10^5^ cells/ml), or A549 (1 × 10^6^ cells/ml) cells, the platelet aggregation rate was monitored by MCM HEMA TRACER 313 M (SSR Engineering, Kagawa, Japan) at 1,000 rpm at 37 °C. In some experiments, the aggregated solution was centrifuged twice at 10,000 *g* for 10 min and filtered (0.2 μm). The supernatant was used as the supernatant of a LSCC-platelet reactant and cultured with naive cells to examine the effect of platelet-derived soluble factors on cell growth.

### Cell growth analysis

A supernatant of LSCC-platelet reactant was collected as described in the Platelet aggregation assay subsection. PC-10/ZsG cells were treated with the supernatant and 0.5% FBS medium to exclude the effect of serum after 1,500 PC-10/ZsG cells were cultured overnight. After 72 hours of incubation, PC-10/ZsG cells were lysed in TENSV buffer (50 mM Tris–HCl pH 7.5, 2 mM EDTA, 100 mM NaCl, 1 mM Na_3_VO_4_, 1% NP-40). The cell viability of the PC-10/ZsG was calculated from ZsGreen fluorescence. Cell growth between PC-10 and PC-10 ΔPDPN or between A549/Neo and A549/PDPN was measured by performing a CellTiter-Glo luminescent cell viability assay (Promega, Madison, WI, USA) according to the manufacturer’s protocols. Cell viability, except in the above case, was determined by Cell Proliferation ELISA, BrdU (Roche Diagnostics, Basel, Switzerland) according to the manufacturer’s protocols. The detection of luminescence or fluorescence was measured by TriStar LB941 Multimode Microplate Reader (Berthold Technologies, Bad Wildbad, Germany).

### Immunofluorescent and immunohistochemistry of *ex vivo* tumours

For immunofluorescent, the cryosection (10 μm) was fixed with 4% paraformaldehyde, and pemeabilized with 0.1% Triton X-100. After blocking with 1% bovine serum albumin, antibodies to PDPN (clone: D2-40, DAKO, Glostrup, Denmark) and Fibrinogen (1:100) (Abcam) were incubated overnight. The slides were subsequently stained with secondary antibodies; 4 μg/ml of Alexa Fluor 594 anti-mouse IgG (H + L) and Alexa Fluor 488 anti-rabbit IgG (H + L) (Thermo Fisher Scientific), respectively. The mounted slides with ProLong Diamond Antifade Mountant with DAPI (Thermo Fisher Scientific) were examined using BioRevo BZ-9000 (Keyence, Osaka, Japan). For immunohistochemistry, the cryosection (10 μm) was fixed in ice-cold acetone and endogenous peroxidase was blocked with 0.3% hydrogen peroxide in methanol. After blocking with 10% goat serum, an antibody to CD41 (1:100) (clone: MWReg30, GeneTex, Irvine, CA, USA) was incubated overnight. Colouring was performed with Anti-Rat Ig HRP Detection Kit (BD Pharmingen). Mayer’s hematoxylin solution (Wako) was used for counterstain. All images were taken with BioRevo BZ-9000 (Keyence). CD41-positive area was analysed by BZ-II Analyzer (Keyence).

### Phospho-RTK analysis

PC-10 cells starved for 24 hours were incubated with a supernatant of a PC-10-platelet reactant. After incubation for 2.5 min, the reaction was stopped with ice-cold phosphate buffer saline (PBS) immediately. The cell lysate (300 μg) was used for the Proteome Profiler Human Phospho-RTK Array Kit (R&D Systems, Minneapolis, MN) to analyze the phosphorylation status of PC-10 cells as according to the manufacturer’s protocols. The signal intensity of phosphorylation was detected by using LAS-3000 mini (Fujifilm) and quantified by using NIH ImageJ software (National Institutes of Health, Bethesda, MD).

### ELISA

A supernatant of PC-10-platelet reactant or PC-10 ΔPDPN-platelet reactants was prepared as described in the platelet aggregation assay subsection. Murine EGF concentration in the supernatant was measured by using an EGF Mouse ELISA Kit (Abcam) according to the manufacturer’s protocols.

### Mouse experiment

PC-10 and PC-10 ΔPDPN cells (1 × 10^7^) in Hank’s Balanced Salt Solution (HBSS) were subcutaneously injected into 4- or 5-week-old female BALB/c-nu/nu (Charles River Laboratories, Yokohama, Japan). After 23 days of cell injection, the mice were euthanized and tumour weights were measured.

For therapeutic experiments, PC-10 cells (5 × 10^6^) in HBSS were subcutaneously injected into 4-or 5-week-old female BALB/c-nu/nu (Charles River Laboratories). After 15 days of cell injection, PC-10 tumour-bearing mice were dosed orally with 50 mg/kg/day erlotinib (LKT Laboratories, Inc., Minnesota, America) for 3 weeks. Clopidogrel sulphate (LC Laboratories, Inc., Alabama, America) was dissolved in drinking water with 0.003% HCl (equal to orally dosed approximately 25 mg/kg/day) for 18 days. When designated treatments were completed, the animals were euthanized and tumours were resected. ChMS-1 antibody treatment was performed as previously described^5^.

A549/Neo and A549/PDPN cells (5 × 10^6^) in HBSS were subcutaneously injected into 4- or 5-week old female BALB/c-nu/nu (Charles River Laboratories). After 22 days of cell injections, the mice were euthanized and tumour weights were measured.

All animal procedures were performed using protocols approved by the Japanese Foundation for Cancer Research Animal Care and Use Committee in accordance with the relevant guidelines and regulations.

### Statistical analysis

Student’s *t*-test or the Mann–Whitney *U*-test was performed to determine the statistical significance of comparisons. Statistical significance was assumed for *P < 0.05 or **P < 0.01. All statistical tests were two-sided.

## Electronic supplementary material

Supplementary Figures

## References

[1] Menter DG (2014). Platelets and cancer: A casual or causal relationship: Revisited. Cancer Metastasis Rev..

[2] Gay LJ, Felding-Habermann B (2011). Contribution of platelets to tumour metastasis. Nat. Rev. Cancer.

[3] Stone RL (2012). Paraneoplastic thrombocytosis in ovarian cancer. N. Engl. J. Med..

[4] Haemmerle M (2016). FAK regulates platelet extravasation and tumor growth after antiangiogenic therapy withdrawal. J. Clin. Invest..

[5] Takagi S (2013). Platelets promote tumor growth and metastasis via direct interaction between Aggrus/podoplanin and CLEC-2. PLoS One.

[6] Labelle M, Begum S, Hynes RO (2014). Platelets guide the formation of early metastatic niches. Proc. Natl. Acad. Sci. USA.

[7] Miyashita T (2015). Metastasis-promoting role of extravasated platelet activation in tumor. J. Surg. Res..

[8] Takagi S, Takemoto A, Takami M, Oh-Hara T, Fujita N (2014). Platelets promote osteosarcoma cell growth through activation of the platelet-derived growth factor receptor-Akt signaling axis. Cancer Sci..

[9] Fujita N, Takagi S (2012). The impact of Aggrus/podoplanin on platelet aggregation and tumour metastasis. J. Biochem..

[10] Kato Y (2003). Molecular identification of Aggrus/T1alpha as a platelet aggregation-inducing factor expressed in colorectal tumors. J. Biol. Chem..

[11] Kato Y (2005). Enhanced expression of Aggrus (T1alpha/podoplanin), a platelet-aggregation-inducing factor in lung squamous cell carcinoma. Tumour Boil.

[12] Kato Y (2004). Aggrus: a diagnostic marker that distinguishes seminoma from embryonal carcinoma in testicular germ cell tumors. Oncogene.

[13] Kunita A, Kashima TG, Ohazama A, Grigoriadis AE, Fukayama M (2011). Podoplanin is regulated by AP-1 and promotes platelet aggregation and cell migration in osteosarcoma. Am. J. Pathol..

[14] Takagi S (2014). Expression of Aggrus/podoplanin in bladder cancer and its role in pulmonary metastasis. Int. J. Cancer.

[15] Kimura N, Kimura I (2005). Podoplanin as a marker for mesothelioma. Pathol. Int..

[16] Bertozzi CC (2010). Platelets regulate lymphatic vascular development through CLEC-2-SLP-76 signaling. Blood.

[17] Uhrin P (2010). Novel function for blood platelets and podoplanin in developmental separation of blood and lymphatic circulation. Blood.

[18] Herzog BH (2013). Podoplanin maintains high endothelial venule integrity by interacting with platelet CLEC-2. Nature.

[19] Kunita A (2007). The platelet aggregation-inducing factor aggrus/podoplanin promotes pulmonary metastasis. Am. J. Pathol..

[20] Suzuki-Inoue K (2007). Involvement of the snake toxin receptor CLEC-2, in podoplanin-mediated platelet activation, by cancer cells. J. Biol. Chem..

[21] Hsieh JC-H (2015). Prognostic value of circulating tumor cells with podoplanin expression in patients with locally advanced or metastatic head and neck squamous cell carcinoma. Head & neck.

[22] Atsumi N (2008). Podoplanin, a novel marker of tumor-initiating cells in human squamous cell carcinoma A431. Biochem. Biophys. Res. Commun..

[23] Wicki A (2006). Tumor invasion in the absence of epithelial-mesenchymal transition: podoplanin-mediated remodeling of the actin cytoskeleton. Cancer Cell.

[24] Nakashima Y (2013). Podoplanin is expressed at the invasive front of esophageal squamous cell carcinomas and is involved in collective cell invasion. Cancer Sci..

[25] Mishima K (2006). Increased expression of podoplanin in malignant astrocytic tumors as a novel molecular marker of malignant progression. Acta Neuropathol..

[26] Hisakane K (2016). Unique intravascular tumor microenvironment predicting recurrence of lung squamous cell carcinoma. J. Cancer Res. Clin. Oncol..

[27] Rahadiani N (2010). Tumorigenic role of podoplanin in esophageal squamous-cell carcinoma. Ann. Surg. Oncol..

[28] Mei Y (2014). Ebp1 activates podoplanin expression and contributes to oral tumorigenesis. Oncogene.

[29] Ran FA (2013). Genome engineering using the CRISPR-Cas9 system. Nat. Protoc..

[30] Foster CJ (2001). Molecular identification and characterization of the platelet ADP receptor targeted by thienopyridine antithrombotic drugs. J. Clin. Invest..

[31] The Cancer Genome Atlas Research Network. Comprehensive genomic characterization of squamous cell lung cancers. *Nature* **489**, 519–525 (2012). 10.1038/nature11404 PMC3466113 22960745

[32] Rosell R (2012). Erlotinib versus standard chemotherapy as first-line treatment for European patients with advanced EGFR mutation-positive non-small-cell lung cancer (EURTAC): A multicentre, open-label, randomised phase 3 trial. Lancet Oncol..

[33] Togashi Y, Hayashi H, Nakagawa K, Nishio K (2014). Clinical utility of Erlotinib for the treatment of non-small-cell lung cancer in Japanese patients: Current evidence. Drug. Des. Devel. Ther.

[34] Achille M, Gallegos-Ruiz M, Giaccone G, Soria J-C (2006). Response to erlotinib in first-line treatment of non-small-cell lung cancer in a white male smoker with squamous-cell histology. Clin. lung cancer.

[35] Neal DE (1990). The epidermal growth factor receptor and the prognosis of bladder cancer. Cancer.

[36] Dreux AC, Lamb DJ, Modjtahedi H, Ferns GAA (2006). The epidermal growth factor receptors and their family of ligands: Their putative role in atherogenesis. Atherosclerosis.

[37] Liska D, Chen CT, Bachleitner-Hofmann T, Christensen JG, Weiser MR (2011). HGF rescues colorectal cancer cells from EGFR inhibition via MET activation. Clin. Cancer Res..

[38] Nakamura T, Teramoto H, Ichihara A (1986). Purification and characterization of a growth factor from rat platelets for mature parenchymal hepatocytes in primary cultures. Proc. Natl. Acad. Sci. USA.

[39] Inoue H (2012). Podoplanin promotes cell migration via the EGF-Src-Cas pathway in oral squamous cell carcinoma cell lines. J. Oral Sci..

[40] Ozaki Y, Suzuki-Inoue K, Inoue O (2013). Platelet receptors activated via mulitmerization: Glycoprotein VI, GPIb-IX-V, and CLEC-2. J. Thromb. Haemost..

[41] Watson AA (2009). The platelet receptor CLEC-2 is active as a dimer. Biochemistry.

[42] Hughes CE (2010). CLEC-2 activates Syk through dimerization. Blood.

[43] Navarro-Núñez L (2015). Platelet adhesion to podoplanin under flow is mediated by the receptor CLEC-2 and stabilised by Src/Syk-dependent platelet signalling. Thromb. Haemost..

[44] Chang Y-W (2015). Identification of a novel platelet antagonist that binds to CLEC-2 and suppresses podoplanin-induced platelet aggregation and cancer metastasis. Oncotarget.

[45] Mitrugno A, Williams D, Kerrigan SW, Moran N (2013). A novel and essential role for Fc RIIa in cancer cell induced platelet activation. Blood.

[46] Chiodoni C (2006). Triggering CD40 on endothelial cells contributes to tumor growth. J. Exp. Med..

[47] Zhang X, Ran Y (2015). Prognostic role of elevated platelet count in patients with lung cancer: a systematic review and meta-analysis. Int. J. Clin. Exp. Med..

[48] Pedersen LM, Milman N (1996). Prognostic significance of thrombocytosis in patients with primary lung cancer. Eur. Respir. J.

[49] Hess PR (2014). Platelets mediate lymphovenous hemostasis to maintain blood-lymphatic separation throughout life. J. Clin. Invest..

[50] Miyata K (2014). Suppression of Aggrus/podoplanin-induced platelet aggregation and pulmonary metastasis by a single-chain antibody variable region fragment. Cancer Med..

