# Supplementary material for: Podoplanin enhances lung cancer cell growth in vivo by inducing platelet aggregation
Source: Sci Rep. 2017 Jun 22;7:4059. doi: 10.1038/s41598-017-04324-1 (PMC5481446; doi:10.1038/s41598-017-04324-1)
Supplement: Supplementary file 1 — Supplementary Figures [file 41598_2017_4324_MOESM1_ESM.pdf]

Supplementary Information

Podoplanin enhances lung cancer cell growth *in vivo* by inducing platelet aggregation

Kenichi Miyata, Ai Takemoto, Sakae Okumura, Makoto Nishio, and Naoya Fujita

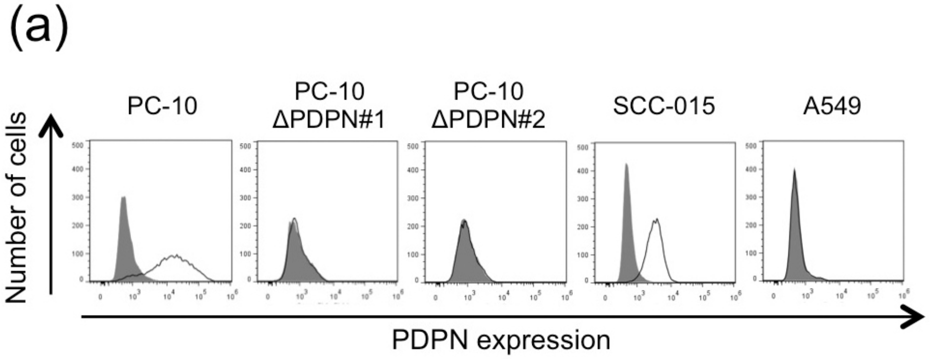

Supplementary Figure S1. Flow cytometric analysis of podoplanin expression in lung cancers. (**a**) The expression of podoplanin was detected by flow cytometric analysis in lung cancer cells, as described in Fig.1a. All cells were treated with control mouse IgG (grey area) or MS-1 antibody (bold line). (**b**) The expression of podoplanin in A549 cells that had been transfected with pcDNA3-mock (A549/Neo) or pcDNA3-podoplanin (A549/PDPN) plasmid was detected by flow cytometric analysis. All cells were treated with control mouse IgG (grey area) or MS-1 antibody (bold line).

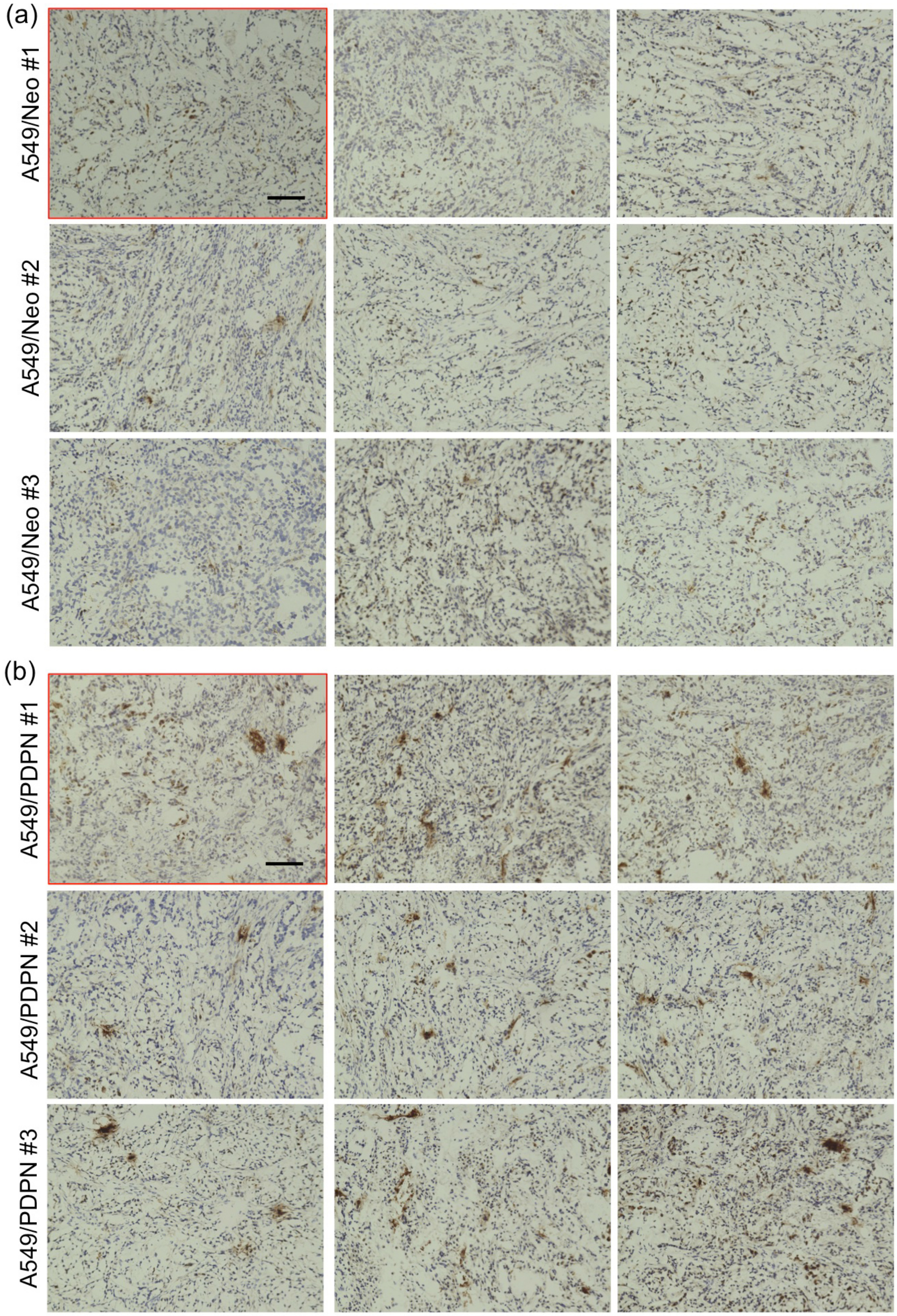
(a)
A549/Neo #1
A549/Neo #2
A549/Neo #3
(b)
A549/PDPN #1
A549/PDPN #2
A549/PDPN #3

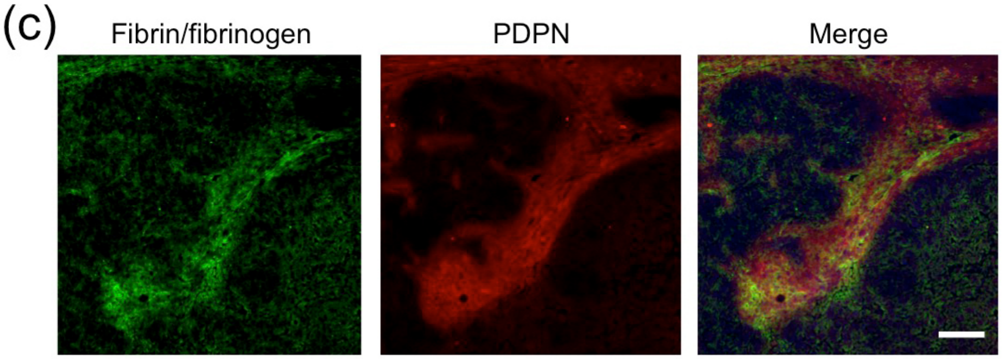

Supplementary Figure S2. PDPN-mediated platelet aggregation (PMPA) was occurred in xenografted PDPN-positive tumour. (**a, b**) A549 cells that had been transfected with pcDNA3-mock (A549/Neo, **a**) or pcDNA3-podoplanin (A549/PDPN, **b**) plasmid were subcutaneously injected into BALB/c-nu/nu mice (Charles River Laboratories, Yokohama, Japan). After 14 days of tumour inoculation, the tumours were extracted and stained with antibodies to a platelet-specific marker CD41. Three independent fields of each tumour section were photographed. The left upper picture highlighted in red was identical to the picture in Figure 2a. Scale bar: 100 μm. (**c**) PC-10 cells were subcutaneously injected into 4- or 5-week-old female NOD.CB17-*Prkdc*^{scid}/J mice (Charles River Laboratories). After 3 weeks of tumour injection, the tumours were extracted and stained with antibodies to Fibrin/fibrinogen (green) and PDPN (red) as described in Methods section. Representative picture was shown. Scale bar: 100 μm.

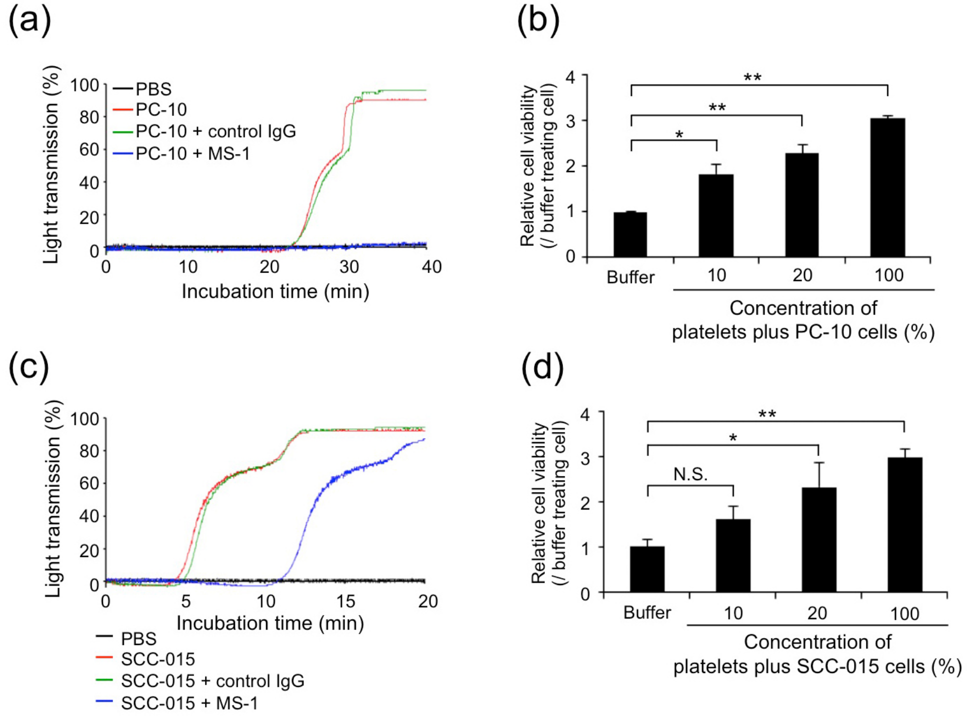

Supplementary Figure S3. Effect of podoplanin on platelet aggregation in LSCC cells. (**a, c**) PC-10 (parent) (**a**) and SCC-015 (**c**) cells were incubated with 200 μg/ml of control mouse IgG or MS-1 antibody for 30 minutes on ice. Then, PC-10 (parent, 1 x 10^6 cells/ml) or SCC-015 (2.5 x 10^5 cells/ml) cells were incubated with washed platelet (5 x 10^8/ml) in Tyrode's buffer with 2% platelet-poor plasma (PPP) and 250 μM $CaCl_2$. Light transmission was measured by MCM HEMA TRACER 313M to monitor platelet aggregation rate. (**b**) PC-10 (parent, 1 x 10^6 cells/ml) cells were incubated with washed platelet (5 x 10^8/ml) in Tyrode's buffer with 2 % PPP and 250 μM $CaCl_2$. After incubation for 40 minutes at 1,000 rpm at 37°C, the supernatants were collected as described in the Materials and Methods. The supernatant was added to PC-10/ZsG cells. After incubation for 72 hours under 0.5% FBS condition, the cell viability of the PC-10/ZsG was calculated from ZsGreen fluorescence. All data are shown as means ± SD of triplicate experiments. *$P < 0.05$ or **$P < 0.01$ by Mann–Whitney *U*-test. (**d**) SCC-015 (2.5 x 10^5 cells/ml) cells were incubated with washed platelet (5 x 10^8/ml) in Tyrode's buffer with 2% PPP and 250 μM $CaCl_2$. After incubation for 20 minutes at 1000 rpm at 37°C, these supernatants were collected as described in the Materials and Methods. The supernatant was added to SCC-015 cells. After incubation for 72 hours under 0.5% FBS condition, the cell growth was determined by Cell Proliferation ELISA, BrdU. All data are shown as means ± SD of triplicate experiments. *$P < 0.05$ or **$P < 0.01$ by Mann–Whitney *U*-test. N.S.: Not significant.

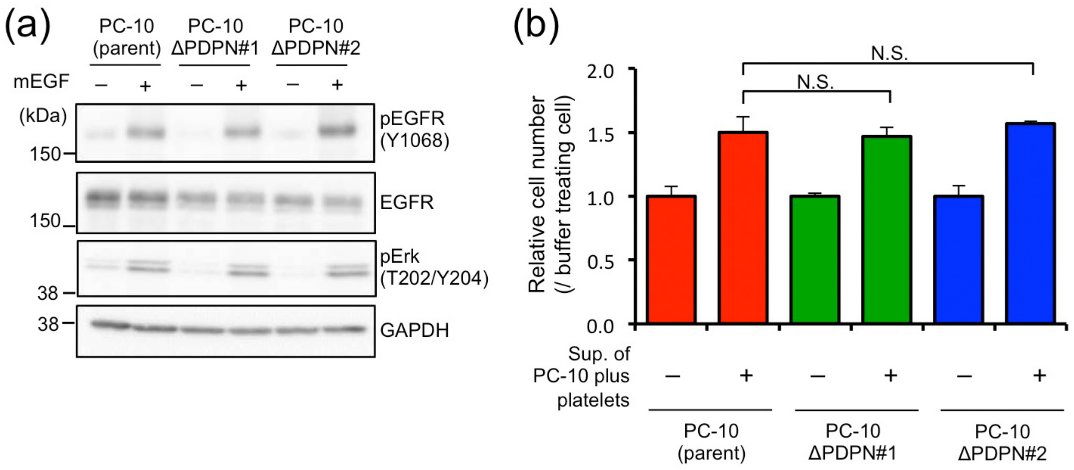

Supplementary Figure S4. Podoplanin does not contribute to activation of EGFR signal in the cells. (**a**) PC-10 (parent), PC-10 ΔPDPN#1 and PC-10 ΔPDPN#2 cells starved for 24 hours were incubated with 5 ng/ml murine epidermal growth factor. After incubation for 2.5 minutes, the reaction was stopped with ice-cold PBS immediately. The cell lysates were analysed for Western blot. (**b**) Supernatants were collected from platelets incubated with PC-10 (parent) cells. Then, PC-10 (parent), PC-10 ΔPDPN#1 and PC-10 ΔPDPN#2 cells were cultured with the collected supernatants under 0.5% FBS condition. After 72 hours, the relative cell number was determined by Cell Proliferation ELISA, BrdU. All data are shown as means ± SD of triplicate experiments. N.S., Not significant by Mann–Whitney *U*-test.

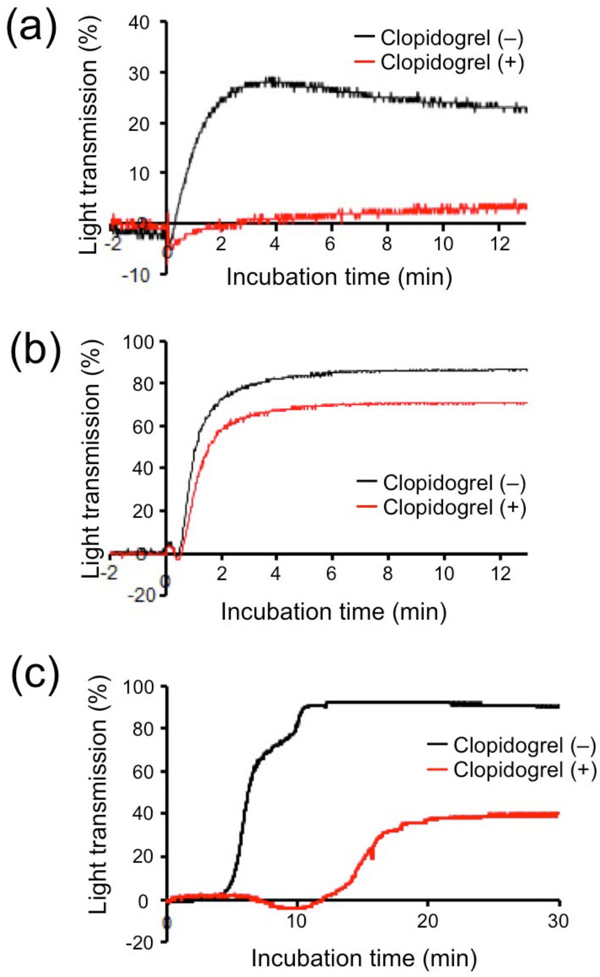

Supplementary Figure S5. The antiplatelet agent clopidogrel suppresses platelet aggregation. (**a-c**) Jcl:ICR mice were orally treated with 25 mg/kg/day clopidogrel for 3 days. Then an additional treatment of 25 mg/kg of clopidogreg was done 1.5 hours prior to collect blood. Washed platelets were collected as described in the Materials and Methods. Then, 100 μM adenosine diphosphate (**a**) or 10 μg/ml of collagen (**b**) was added to the washed platelets (5 x 10^8/ml) containing 250 μM $CaCl_2$ in modified Tyrode's buffer after 2 minutes of preincubation. PC-10 cells (1 x 10^6 cells/ml) (**c**) were incubated with washed platelets (5 x 10^8 /ml) containing 250 μM $CaCl_2$ and 2% platelet-poor plasma in modified Tyrode's buffer. The light transmission was measured by MCM HEMA TRACER 313M to monitor platelet aggregation rate.

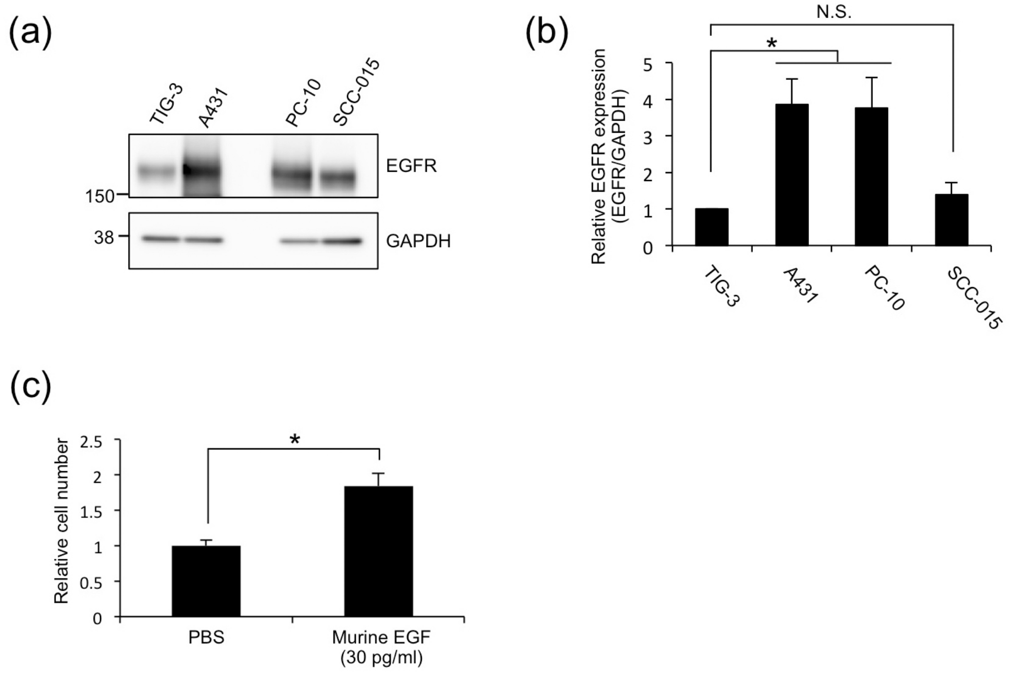

Supplementary Figure S6. EGF stimulated the growth of SCC-015 cells that expressed normal level EGFR. (**a**) Western blot analysis of EGFR expression. The cell lysate of TIG-3, A431, PC-10 and SCC-015 cells were electrophoresed and immunoblotted with antibodies to EGFR or GAPDH. (**b**) EGFR expression level in TIG-3, A431, PC-10 and SCC-015 cells was quantified using NIH ImageJ software (National Institutes of Health, Bethesda, MD). All data are shown as mean ± SEM of independent triplicate experiments. *$P < 0.05$ by Mann–Whitney *U*-test. N.S.: Not significant. (**c**) SCC-015 cells were incubated with 30 pg/ml of murine EGF under 0.5% FBS condition. After 72-hour incubation, cell number was determined by Cell Proliferation ELISA, BrdU (Roche Diagnostics, Basel, Switzerland). Relative cell number was normalized by PBS-treated cells as the control. All data are shown as means ± SD of triplicate experiments. *$P < 0.05$ by Student's *t*-test.

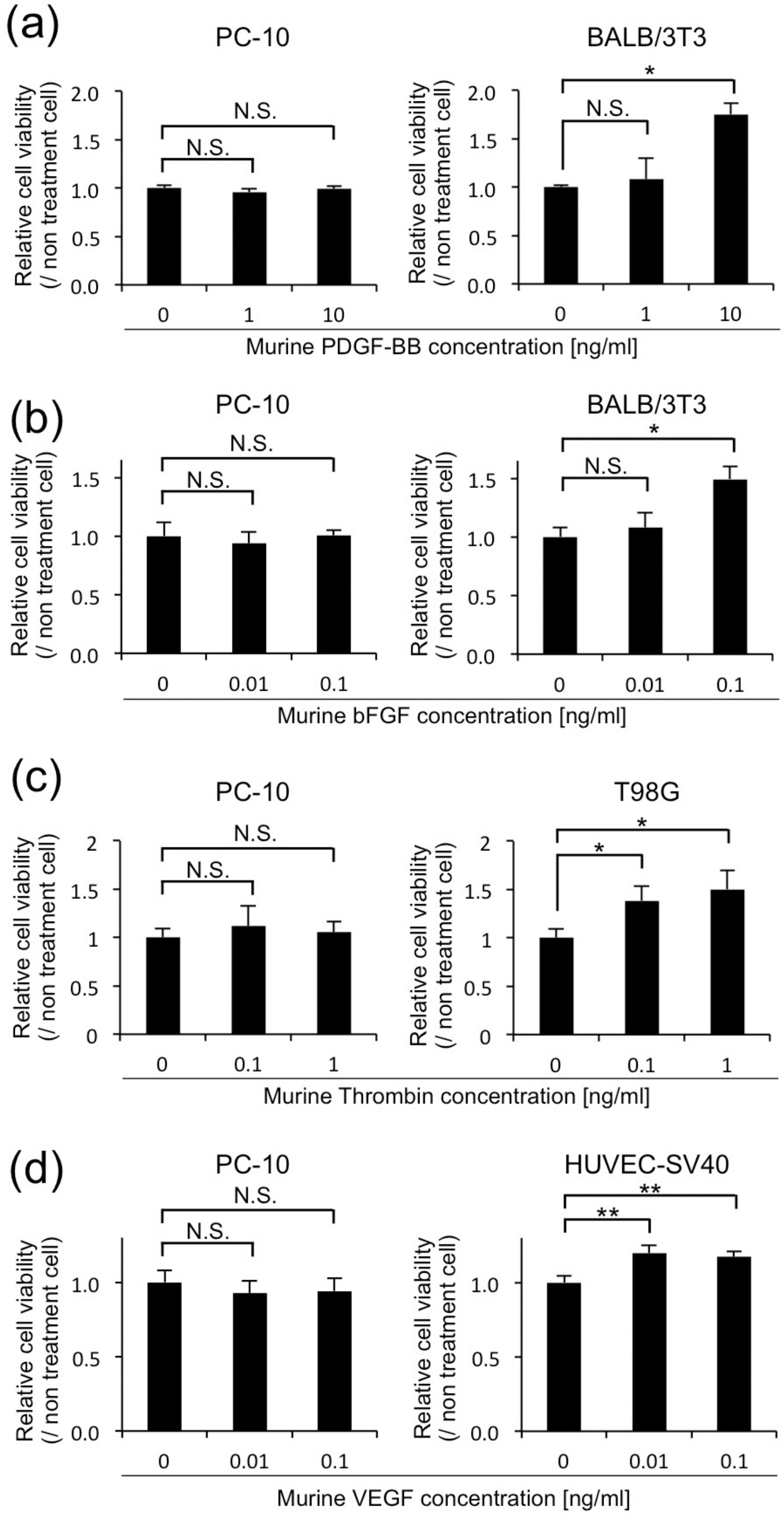

(a)
PC-10
BALB/3T3
Relative cell viability (/ non treatment cell)
N.S.
N.S.
*
N.S.
2.0
1.5
1.0
0.5
0.0
0
1
10
Murine PDGF-BB concentration [ng/ml]
(b)
PC-10
BALB/3T3
N.S.
N.S.
*
N.S.
1.5
1.0
0.5
0.0
0
0.01
0.1
Murine bFGF concentration [ng/ml]
(c)
PC-10
T98G
N.S.
N.S.
*
*
2
1.5
1
0.5
0
0
0.1
1
Murine Thrombin concentration [ng/ml]
(d)
PC-10
HUVEC-SV40
N.S.
N.S.
**
**
1.0
0.5
0.0
0
0.01
0.1
Murine VEGF concentration [ng/ml]

Supplementary Figure S7. The effects of platelet-derived factors on PC-10 cell growth. (**a**) PC-10 (left) and BALB/3T3 (right) cells were incubated with the indicated concentration of recombinant murine PDGF-BB (PeproTech, Rocky Hill, NJ, USA). After incubation for 72 hours under 0.5% FBS condition, the cell growth was estimated using CellTiter-Glo luminescence cell viability assay kit. BALB/3T3 cells were used as PDGF-BB-responsive positive control cells. (**b**) PC-10 (left) and BALB/3T3 (right) cells were incubated with the indicated concentration of recombinant murine FGF-basic (bFGF, PeproTech). After incubation for 72 hours under 0.5% FBS condition, the cell growth was estimated using CellTiter-Glo luminescence cell viability assay kit. BALB/3T3 cells were used as bFGF-responsive positive control cells. (**c**) PC-10 (left) and T98G (right) cells were incubated with the indicated concentration of murine thrombin (Molecular Innovations, Novi, MI, USA). After incubation for 72 hours under 0.5% FBS condition, the cell growth was estimated using CellTiter-Glo luminescence cell viability assay kit. T98G cells were used as thrombin-responsive positive control cells. (**d**) PC-10 (left) and HUVEC-SV40 (right) cells were incubated with the indicated concentration of recombinant murine $VEGF_{165}$ (PeproTech). After incubation for 72 hours under 0.5% FBS condition, the cell growth was estimated using CellTiter-Glo luminescence cell viability assay kit. HUVEC-SV40 cells were used as VEGF-responsive positive control cells.

All data are shown as means ± SD of sextuplicate experiments. *$P < 0.05$ or **$P < 0.01$ by Mann–Whitney U-test. N.S.: Not significant.

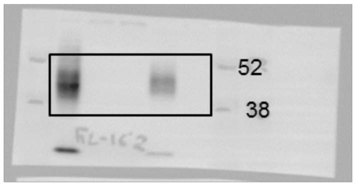

Fig. 1a PDPN
(Optimal exposure)

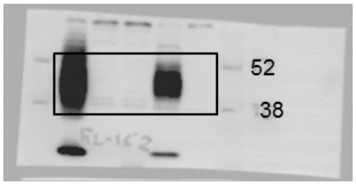

Fig. 1a PDPN
(High exposure)

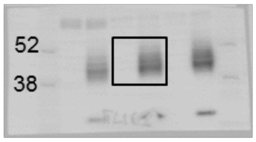

Fig. 1d PDPN
(Optimal exposure)

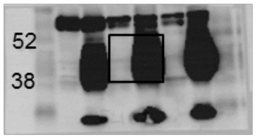

Fig. 1d PDPN
(High exposure)

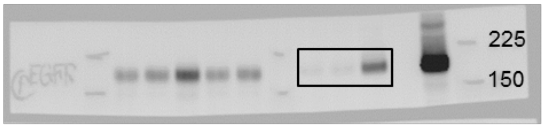

Fig. 4b pEGFR (Optimal exposure)

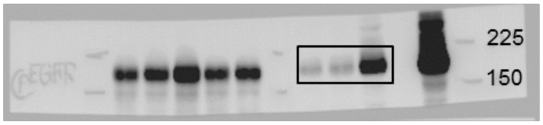

Fig. 4b pEGFR (High exposure)

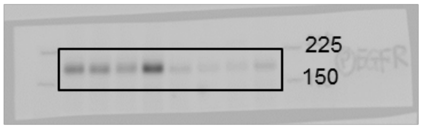

Fig. 5b pEGFR
(Optimal exposure)

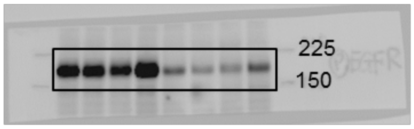

Fig. 5b pEGFR
(High exposure)

Supplementary Figure S8. For high-contrast of gels (Figures 1a, 1d, 4b and 5b), multiple exposure images of full-length blots were presented.
